# Fabrication of Fluorine and Nitrogen-Based Flame Retardants Containing Rigid Polyurethane Foam with Improved Hydrophobicity and Flame Retardancy

**DOI:** 10.1021/acsomega.5c00603

**Published:** 2025-04-22

**Authors:** Merve Nizam, Tuba Çakır Çanak, İbrahim Ersin Serhatlı

**Affiliations:** † Department of Chemistry, Istanbul Technical University, 34469 Maslak, Istanbul, Turkey; ‡ Department of Polymer Science and Technology, Institute of Science and Technology, Istanbul Technical University, 34469 Maslak, Istanbul, Turkey

## Abstract

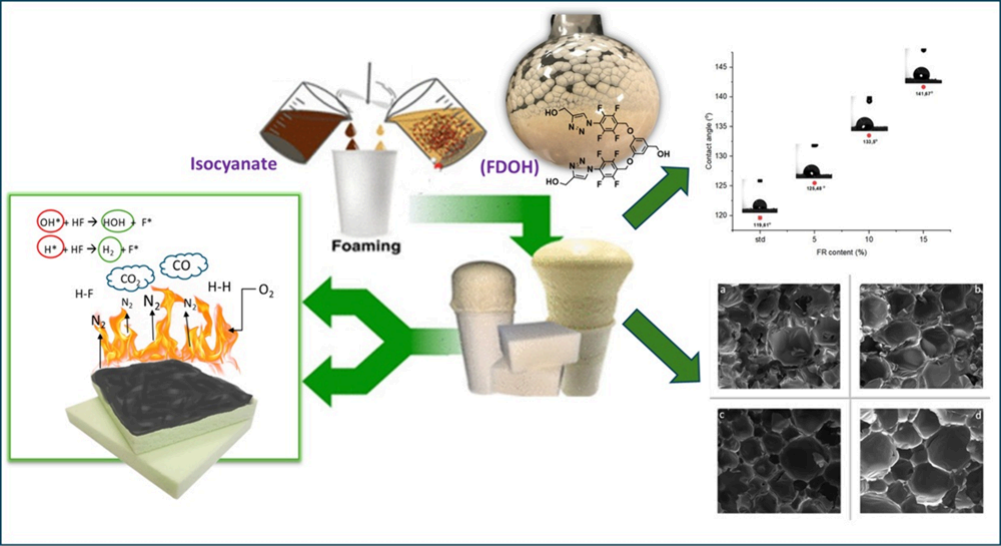

In this work, a novel flame retardant containing fluorine and nitrogen was synthesized by using a copper-catalyzed azide–alkyne cycloaddition reaction, resulting in a hydroxyl-terminated compound with a triazole structure. This flame retardant was added to rigid polyurethane foam (RPUF) formulations in varying percentages (5, 10, and 15%) in order to chemically bond to isocyanates in the foam. Modified RPUF samples were analyzed for structural properties (FTIR), flammability (cone calorimetry, LOI, TGA), thermal conductivity, morphology (SEM), mechanical properties, and hydrophobicity. The results indicated significant improvements in flame retardancy with higher LOI values and lower peak heat release rates. The flame retardant chemically bonded to the foam, maintaining its mechanical integrity and thermal insulation and increasing hydrophobicity due to its fluorine content. This study highlights the potential of fluorine- and nitrogen-containing flame retardants for future fire-resistant materials.

## 1 Introduction

Polyurethane is a polymer of the raw material with the highest volume that cannot be characterized by its basic structure. Instead, polyurethane represents a class of polymers, and any polymer with urethane repeat units is classified as a polyurethane, regardless of other functional structures or the incorporated polymer structure. Thus, a standard polyurethane may include aliphatic and aromatic hydrocarbons, esters, ethers, amides, urea, and isocyanurate groups, in addition to the urethane linkages.^1−4^ Polyurethanes have emerged as one of the crucial categories among polymeric materials. This is primarily due to the wide array of inherently diverse precursor materials, which allows the formulation and synthesis of polyurethane-based materials endowed with a wide spectrum of properties suitable for a multitude of applications.^5−8^ Polyurethane-based polymers were invented in the 1930s. They are characterized by the urethane linkage: −NH–C(=O)–O–.^9−12^ The linkage is formed by the reaction of polyisocyanate isocyanate groups with polyol hydroxyl groups. Polyurethanes are available in both thermoplastic and thermoset forms. Thermoplastic polyurethane is highly durable and exhibits high resistance to abrasion and chemicals. It is commonly used as a foaming material, producing rigid or flexible foams.^13−17^ On the other hand, thermoset polyurethane increases in hardness as the degree of cross-linking increases. Its excellent durability, good traction properties, and resistance to wear make it ideal for applications such as roller skates and shoe heels. Typically, polyurethanes consist of a soft segment derived from either polyester or polyether coupled with a rigid segment based on diisocyanate. Polyurethane foams are produced by the reaction of polyols with polyisocyanates in the presence of blowing agents.^18^ The resulting foam properties are influenced by the molecular weight and functionality of the polyols. Polyisocyanates serve as the connecting agents for polyols. Consequently, urethane and related foams are acknowledged as foundational polymers in the field of materials science. Flexible foams generally have an open-cell structure, while rigid polyurethane foams exhibit a predominantly closed-cell structure (with over 90% closed cells). This closed-cell structure gives excellent thermoinsulation properties to rigid polyurethane foams, making them crucial in various applications. Rigid PU foams are widely used for thermoinsulation in freezers, constructions, storage tanks, pipes, and construction elements like sandwich panels.^19−22^ Most polymers, composed mainly of carbon and hydrogen, are highly flammable because of their chemical nature. Flexible and rigid urethane foams have been associated with significant fire hazards.^23^ The thermal breakdown of polyurethane foams causes the dissociation of the urethane linkage, producing amines through CO_2_ elimination.

Combustion in air produces CO_2_, H_2_O, and various CN-containing compounds, such as HCN, with minimal char formation, which contributes to dense smoke. To overcome the flammability of polyurethane, reactive (for example, brominated polyethers), mainly in the form of alcohols, and nonreactive (for example, antimony trioxide) are used in formulations.^24,25^ Conventional flame retardants for foams include tris(2-chloroethyl) phosphate or tris(1-chloro-2-propyl) phosphate. Other additives commonly used in polyurethane foam applications are phosphorus-based compounds, such as triphenyl phosphate; phosphonates, such as dimethyl propyl phosphonate, ammonium polyphosphate (APP), and phosphaphenanthrene oxide (DOPO derivatives); nitrogen derivatives, such as melamine, melamine cyanurate, and melamine polyphosphate; polymeric silicone derivatives; and inorganic flame retardants, such as magnesium hydroxide and aluminum trihydroxide.^26−30^ Sykam et al. investigated the flame-retardant properties of triazole-based additives in polyurethane foams derived from castor oil. Using a phosphorus- and triazole-functionalized monomer (PTFM), they enhanced the thermal stability of the foams and improved the formation of the char layer during combustion. The protective layer of carbon reduced the rate of oxygen and heat transfer, which decreased the combustion rate. The higher PTFM content increased the yield of the char, reduced the heat release, and achieved a rating of V-1 with 27% LOI, demonstrating the effectiveness of phosphorus and triazole in flame-resistant, environmentally friendly foams.^31−33^

There are several examples of phosphorus-containing polyurethane foam in the literature; however, the synthesized one will be the first for both flame retardancy and hydrophobicity. The main objective of this study was to synthesize a novel triol-based flame retardant to enhance the fire resistance and water repellency of rigid polyurethane foams. To synthesize the target compound, the production of 3,5-bis(perfluorobenzyloxy)benzyl alcohol, followed by the conversion of parafluoro groups into azides, and finally, a copper-catalyzed azide–alkyne cycloaddition reaction with propargyl alcohol, was achieved to yield the triol product. The obtained triol compound was bound to the polyurethane matrix chemically, but not as a flame retardant additive. It was incorporated into rigid polyurethane foam formulation at concentrations of 5, 10, and 15%. It offers long-lasting protection with minimal harmful gas emissions. Additionally, the fluorine content enhances the water resistance of the foam. The obtained rigid polyurethane foams (RPUFs) showed flame-retardant properties and were evaluated with limiting oxygen index (LOI) tests, cone calorimetry, and thermogravimetric analysis (TGA). Mechanical strength and compatibility were assessed by compression tests and scanning electron microscopy (SEM). These properties were compared to those of standard RPUF, which highlighted improvements in flame retardancy, thermal stability, and water resistance.

## 2 Experimental Section

### 2.1 Materials

3,5-Dihydroxybenzyl alcohol (99%, Sigma-Aldrich); pentafluorobenzyl bromide (98%, Alfa Aesar); 18-crown-6 (99%, Merck); anhydrous potassium carbonate (99%, Merck); magnesium sulfate anhydrous (Merck); sodium azide for synthesis (NaN_3_, Merck); *N*,*N*,*N*′,*N*″,*N*″-pentamethyldiethylenetriamine (97%, PMDETA, Sigma-Aldrich); copper(I)bromide (98.0%, Sigma-Aldrich); propargyl alcohol (99%, Sigma-Aldrich); aluminum oxide 90 active neutral (for column chromatography, Merck); sodium sulfate anhydrous (Merck); dichloromethane (JT Baker); hexane (99%, Sigma-Aldrich); diethyl ether (Honeywell); chloroform (for analysis, Merck); and molecular sieves were used. Acetone (99%, Sigma-Aldrich) was dried and distilled. *N*,*N*-dimethylformamide (for analysis, Merck) was dried with 3Å molecular sieves. 400 OH sucrose-based polyether polyol and polymeric methylene diphenyl diisocyanate were purchased from Flokser Textile and Chemistry Company, Istanbul, Turkey.

### 2.2 Synthesis of 3,5-Bis(perfluorobenzyloxy)benzyl Alcohol (FOH)

In the course of this study, a nitrogen-absorbing head was affixed to the center of a three-neck round-bottom flask, which was subsequently placed in a stirrer. After a period of 30 min to allow nitrogen to circulate within the empty flask, a mixture consisting of 0.024 mol (3.42 g) of 99% pure 3,5-dihydroxybenzyl alcohol and 100 mL of distilled acetone was introduced into the flask. After a further half-hour interval to ensure complete dissolution, 0.024 mol (0.64 g) of 18-crown-6, dissolved in 10 mL of acetone, was added to the solution, resulting in a transition to a coffee-brown hue. Subsequent additions included 0.05 mol (7.18 mL) of pentafluorobenzyl bromide, 0.05 mol (7.04 g) of potassium carbonate, and 40 mL of acetone.

After the system was allowed to reach equilibrium under a nitrogen atmosphere for 1 h, the nitrogen connection was removed, and the flask was sealed. Over the course of 4 days, the solution was stirred continuously at high speed, gradually becoming a light pink color. The solvent was then removed by rotary evaporation. Following purification through an extraction process that employed distilled water and dichloromethane (DCM) to eliminate impurities, the DCM phase was dried with magnesium sulfate (MgSO_4_).

The product, FOH, was obtained by crystallization of 50% (v/v) hexane/DCM, and NMR analysis data are given in Table 1.^34^ (Yield = 71.8%) ^1^H NMR (500 MHz, CDCl_3_) δ 6.67, 6.50, 5.11, 4.67, 1.69. ^13^C NMR (126 MHz, CDCl_3_) δ: 159.73, 147.15, 145.12, 144.36, 143.23, 141.19, 138.97, 136.96, 110.32, 106.64, 101.70, 65.38, 57.89. ^19^F NMR (470 MHz, CDCl_3_) δ: −142.32, −152.53, −161.47.

**Table 1:** NMR Elemental Analysis Data

shift (ppm)	integration	multiplicity	assignment
6.67	H	singlet	–CH aromatic (d)
6.50	2H	singlet	–CH aromatic (c)
5.11	4H	singlet	–CH_2_–O (e)
4.67	2H	dublet	–CH_2_–OH (b)
1.69	H	triplet	–OH (a)

### 2.3 Synthesis of (3,5-Bis((4-azido-2,3,5,6-tetrafluorobenzyl)oxy)phenyl)methanol (FOH-N_3_)

Using a three-necked round-bottom flask and an oil bath, a reflux system is set up on a heater. A nitrogen connection is attached to the system. Next, 0.004 mol of FOH is allowed to dissolve thoroughly in 30 mL of dimethylformamide (DMF) for half an hour. Subsequently, 0.016 mol (4 times) of sodium azide was added. The oil bath temperature is set at 60 °C to initiate reflux. After 5 h, the nitrogen connection is disconnected. The system is left in this state overnight. To remove DMF and impurities from the solution, 30 mL of distilled water and 60 mL of diethyl ether are added, and extraction is performed. The diethyl ether phase is collected and dried with magnesium sulfate (MgSO_4_), and then, the solvent is removed using a rotary evaporator. The compound is thoroughly dried in a vacuum oven.^35^ (Yield = 94.9%) ^1^H NMR (500 MHz, CDCl_3_) δ 6.66, 6.49, 5.09, 4.66, 1.72. ^13^C NMR (126 MHz, CDCl_3_) δ: 159.75, 147.02, 145.03, 144.30, 141.91, 139.78, 121.36, 110.63, 106.63, 101.68, 65.38, 57.97. ^19^F NMR (470 MHz, CDCl_3_) δ: −142.78, −151.61.

### 2.4 Synthesis of Flame Retardants Based on Fluorine and Nitrogen (FDOH)

**Figure 1:**
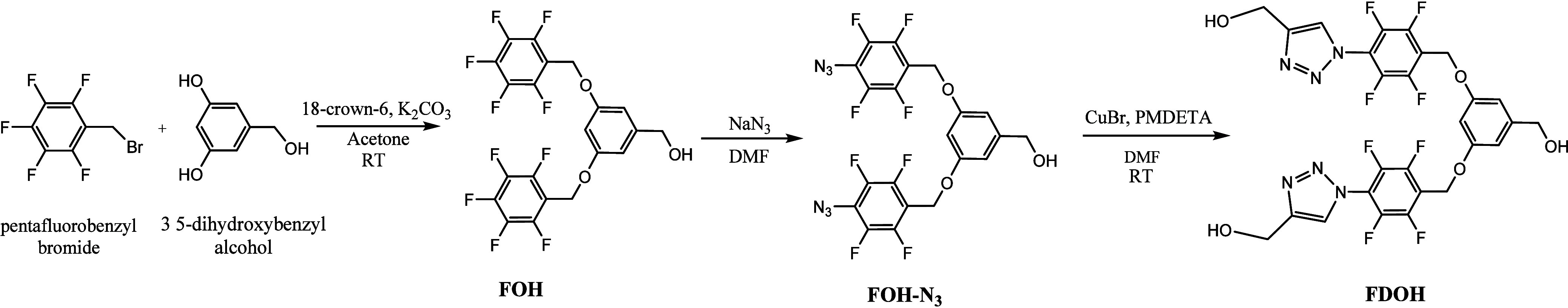
Synthesis of FDOH.

FDOH was synthesized according to the related literature, with some modifications given in Figure 1. Before adding any substances, nitrogen was circulated through a round-bottom flask with three necks for half an hour. After this period, 0.00366 mol of FOH-N_3_ and 30 mL of dry DMF were added to the flask.

Subsequently, 5.5 × 10^–4^ mol of PMDETA (*N*,*N*,*N*′,*N*″,*N*″-pentamethyldiethylenetriamine), 5.5 × 10^–4^ mol of CuBr (copper bromide (I)), and finally, 0.011 mol of propargyl alcohol were added. The addition of CuBr resulted in the solution turning dark green. After the additions were complete, the reaction mixture was stirred at room temperature overnight. Then, the reaction was terminated, and a neutral aluminum oxide column was used to remove CuBr from the solution. The column was washed with chloroform. The collected solution was extracted with water, dried with sodium sulfate, and then completely dried using a rotary evaporator, followed by a vacuum oven. The final product was obtained as a light orange powder.^36,37^ (Yield = 76.2%) ^1^H NMR (500 MHz, CDCl_3_) δ 7.88, 6.64, 6.49, 5.21, 5.08, 4.90, 4.65, 1.25. ^13^C NMR (126 MHz, CDCl_3_) δ: 159.71, 148.50, 146.99, 145.01, 144.36, 141.91, 139.91, 124.51, 121.26, 110.62, 106.58, 101.58, 65.26, 57.94, 56.67. ^19^F NMR (470 MHz, CDCl_3_) δ −142.84, −151.68.

### 2.5 Preparation of Rigid Polyurethane Foams

RPUF samples were prepared using a one-shot process, whereby they were manually mixed at specific amounts, as seen in Table 2, using a high-speed mechanical stirrer. Subsequently, the mixture was rapidly poured into a mold preheated with hot water, with a size of 30 × 30 × 10 cm, and left to cure.

**Table 2:** Chemical Compositions of Polyurethane Samples

polyurethane	isocyanate (g)	polyol (g)	FDOH (g)
standard PU	135	100	
5% FR/PU	135	100	11.75
10% FR/PU	135	100	23.5
15% FR/PU	135	100	35.25

The foams were cut to dimensions compatible with the testing standards after being removed from the molds. The mass quantities of RPUFs prepared with the flame retardant material at 5, 10, and 15% are given below.

### 2.6 Characterization Methods

^1^H NMR, ^13^C NMR, and ^19^F NMR spectra of reaction products in deuterated chloroform (CDCl_3_) were recorded on an Agilent VNMRS 500 MHz spectrometer to characterize the chemical composition of the compounds.

Fourier transform infrared spectra of the three reaction products, neat RPUF, and RPUFs containing the synthesized flame retardant in different percentages were taken with a Thermo Scientific Nicolet FTIR IS 10 spectrometer with a resolution mode of 4 cm^–1^. The average of 16 scans was used for each sample in the range of 4000–600 cm^–1^. Thermal gravity analysis of PU foam samples was performed with a TA Q50 instrument (TGA). Samples of 10–15 mg were placed in a platinum sample pan and heated from 20 to 900 °C under a N_2_ atmosphere at a flow rate of 90 mL/min. The heating rate was set at 20 °C/min, and weight loss and residue were recorded as a function of temperature.

LOI analyses of PU foams were performed with a MARESTEK instrument. LOI analyses of RPUF samples 100 × 10 × 10 mm in size were performed with the MARESTEK instrument according to ISO 4589.

The cell morphology of standard and flame-retardant RPUFs was investigated using a scanning electron microscope. Before SEM analysis, RPUF samples were prepared by cutting them to a 10 mm × 10 mm size. Subsequently, the samples underwent a process of Au–Pd sputter coating to render them electrically conductive. SEM analysis was performed with a Quanta FEG 250, and samples were coated with a Quorum SC 7620 sputter coil.

A compression test was conducted to assess the mechanical properties of RPUF samples. The test was performed using a Shimadzu AGS-J instrument according to the ASTM D1621. The dimensions of the samples are 50 × 50 × 35 mm.

Measurement of thermal conductivity is an important test for materials that are critical in terms of heat for their intended applications. For example, RPUF is a material with very low thermal conductivity, which makes it stand out as an insulation material. A LaserComp heat flow meter, compliant with the ASTM C518 standard, manufactured by TA Instruments, was utilized for the thermal conductivity measurement. For the test, the dimensions of the RPUF samples were 240 × 240 × 35 mm.

The cone calorimetry test is an important method commonly used to determine the combustion behavior of materials. The following parameters were measured using a cone calorimeter instrument in accordance with ASTM E1354 and ISO 5660 standards for standard (blank) and flame-retardant RPUF samples: Heat release rate, total heat release, maximum heat release value, effective heat combustion rate, CO and CO_2_ emissions, mass loss amount, and rate. A specimen size of 100 × 100 × 25 mm was used for all samples.

Contact angle measurements were conducted to investigate the effect of the presence of flame-retardant compounds containing fluorine groups in polyurethane foam at various percentages (5, 10, and 15) on material properties, such as liquid absorption, wettability, and hydrophobicity.

The contact angle was measured using a digital goniometer that captured images of the liquid interface and automatically calculated the angle. The Theta Lite attenuation device belonging to Biolin Scientific was used in the measurements.

## 3 Results and Discussion

### 3.1 Characterization of FDOH

**Figure 2:**
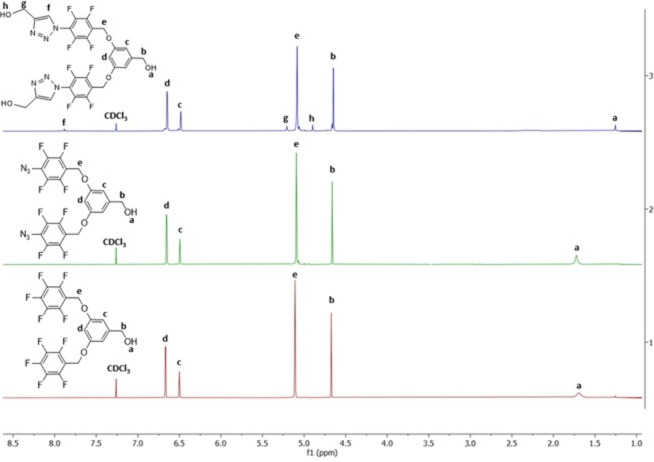
Comparison of ^1^H NMR spectra of FOH (1), FOH-N_3_ (2), and FDOH (3) in CDCl_3_.

**Figure 3:**
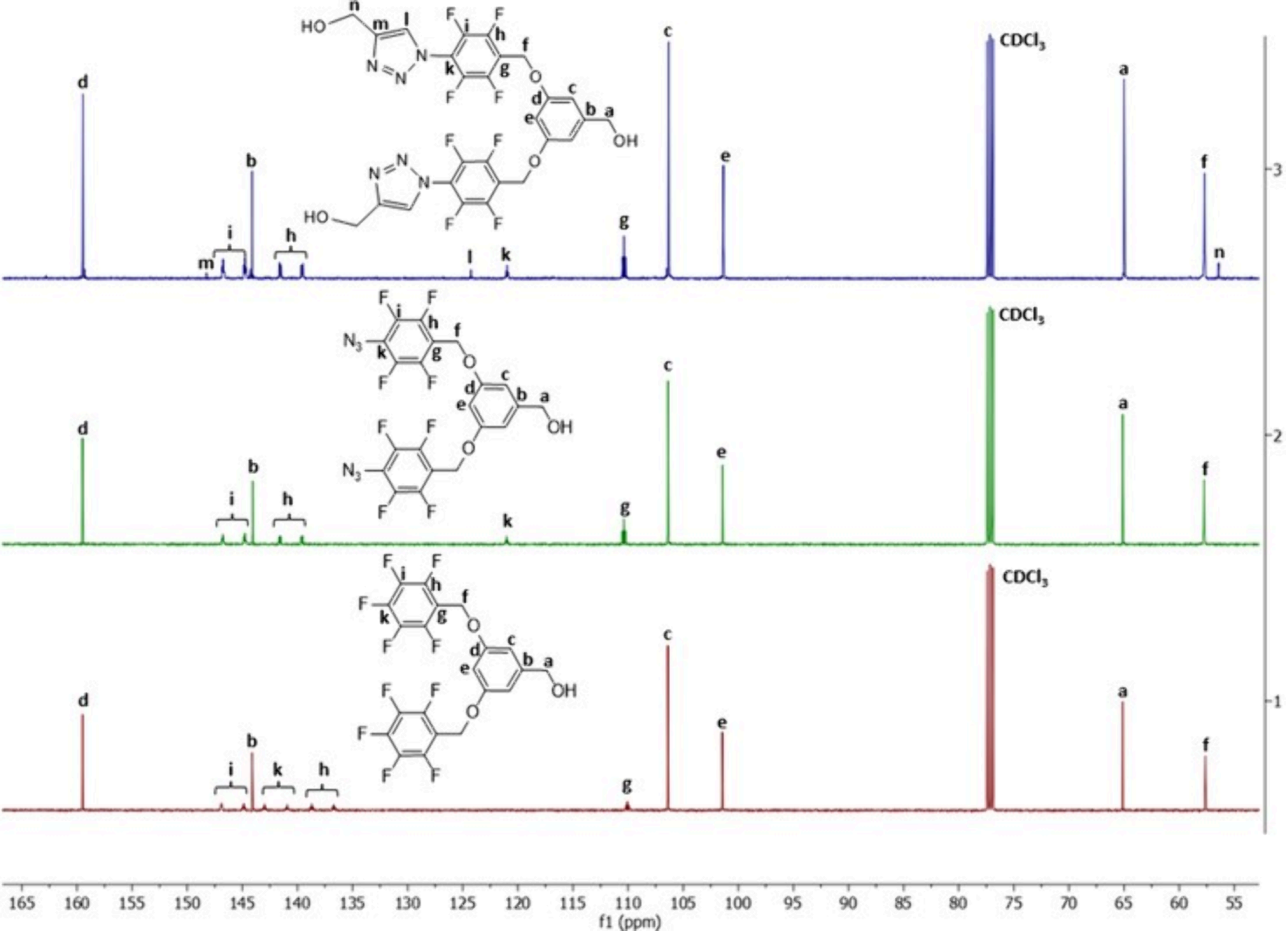
Comparison of ^13^C NMR spectra of FOH (1), FOH-N_3_ (2), and FDOH (3) in CDCl_3_.

**Figure 4:**
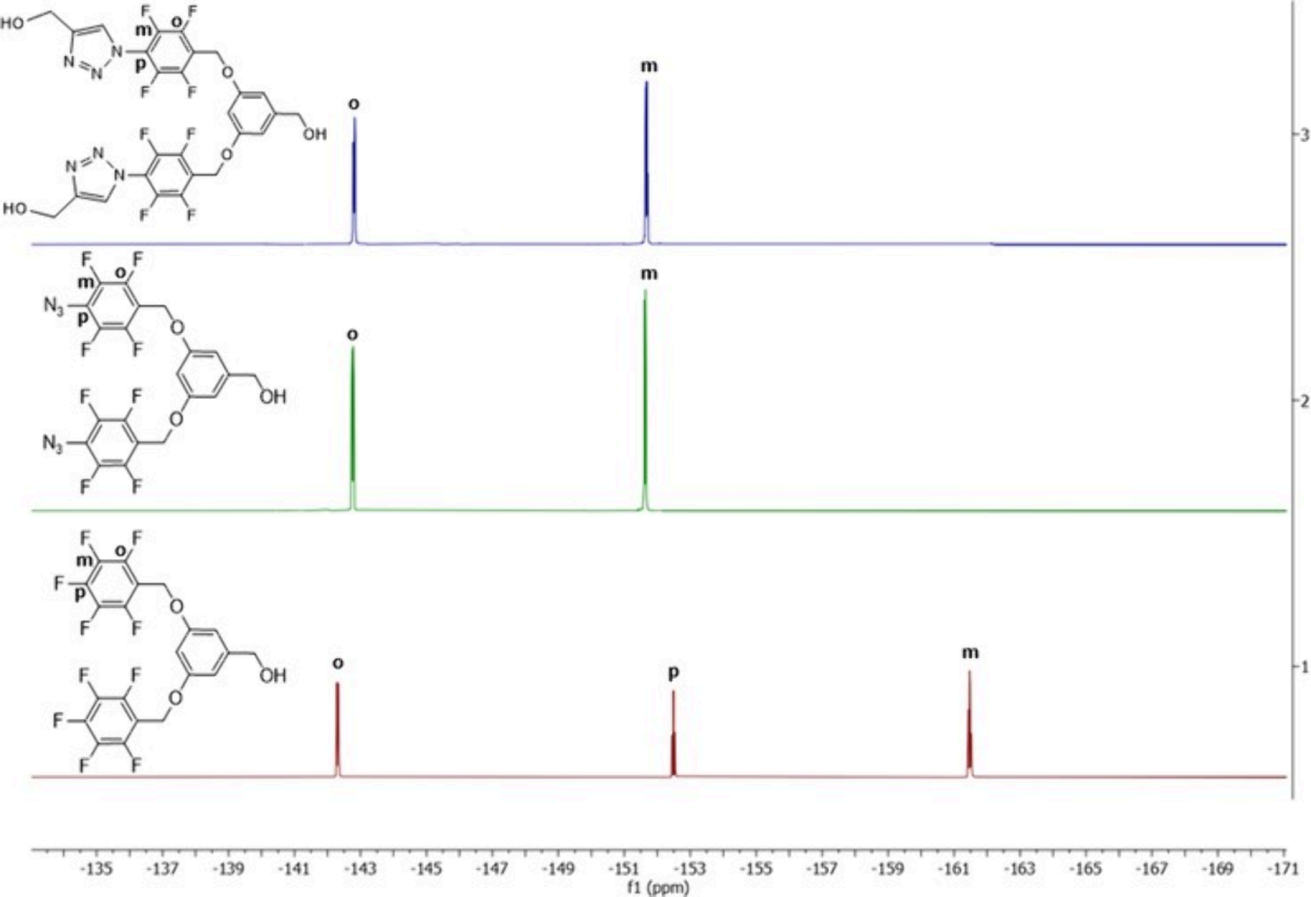
Comparison of ^19^F NMR spectra of FOH (1), FOH-N_3_ (2), and FDOH (3) in CDCl_3_.

**Figure 5:**
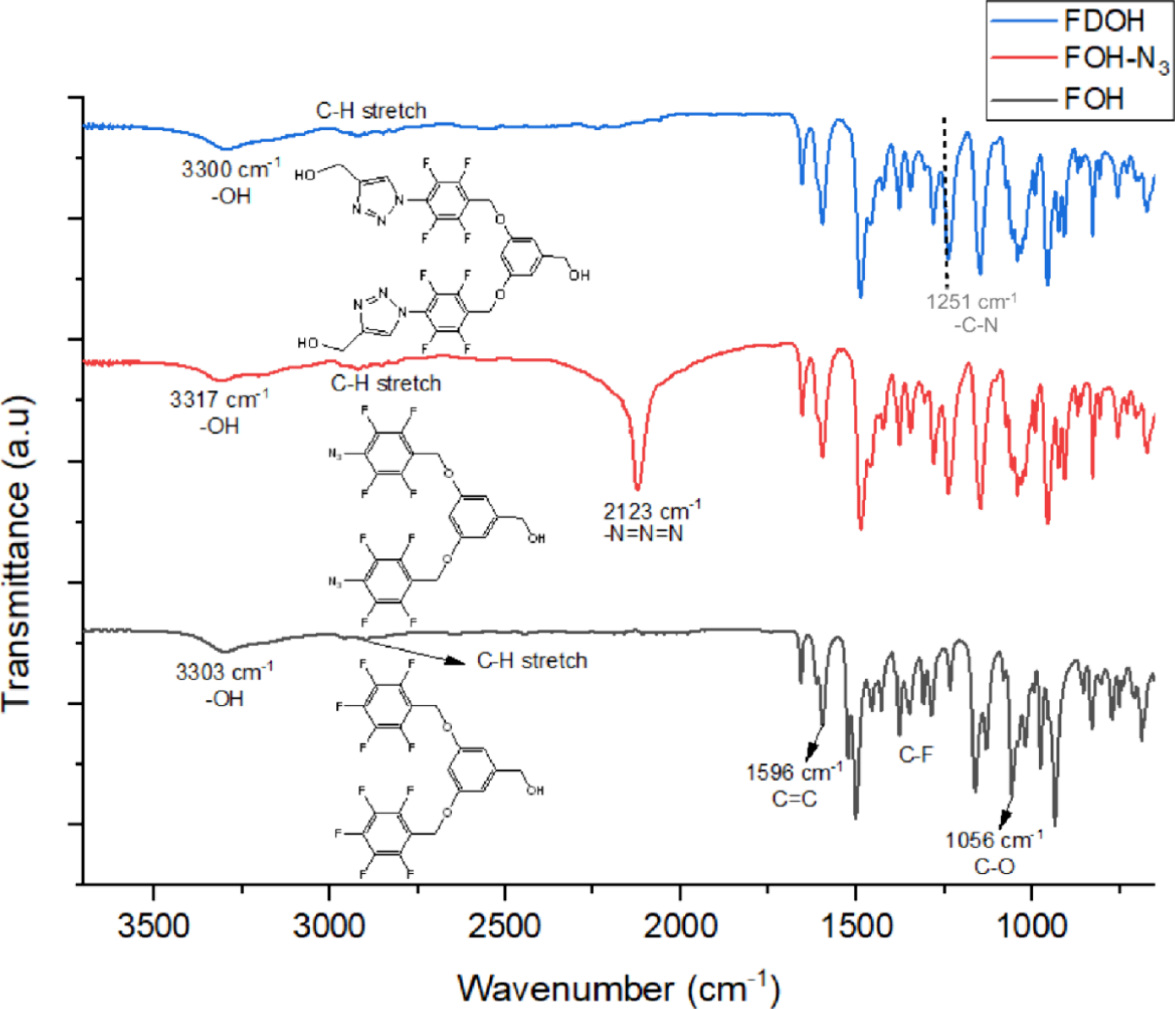
FTIR spectra of FOH, FOH-N_3_, and FDOH.

The structural characterization of all steps of the synthesized flame retardant was investigated by FTIR, ^1^H NMR, ^19^F-NMR, and ^13^C NMR analyses. Examining the proton NMR spectrum in Figure 2 reveals that the substitution of the para-fluorine group with the −N_3_ group did not induce any changes in the proton NMR, as expected. However, the formation of the triazole structure resulted in the emergence of three new peaks in the blue spectrum, appearing at 7.89 (f), 5.22 (g), and 4.91 (h). These new peaks align with values found in literature examples.^35,37^ In contrast to proton NMR, as can be seen in the carbon NMR spectrum in Figure 3, the peak at 141.95 ppm, attributed to the para-fluorine group, disappears upon detachment of fluorine and attachment of the −N_3_ group, and a new peak forms at 121.01 ppm. Subsequently, with the formation of the triazole structure, three new peaks emerge at 148.25 (m), 124.25 (l), and 56.42 (n) ppm. According to the ^19^F NMR analyses of FOH and FOH-N_3_ shown in Figure 4, the disappearance of the intense peak at −152.49 ppm of fluorine in the para position of FOH in the NMR spectrum of the FOH-N_3_ molecule provides further evidence that the para-fluorine groups are separated and replaced by azide (−N_3_) groups. Similarly, in the ^19^F NMR analysis of FDOH, two sharp peaks belonging to the ortho and meta fluorine groups were observed at −142.82 and −151.68 ppm, respectively.^37^ The FTIR analyses and their respective details for each stage of the three-step synthesis can be observed in Figure 5. The FTIR spectrum of the compound with the FOH code revealed key functional groups: a peak at 3303 cm^–1^ signifies −OH, a stretch around 2900 cm^–1^ indicates C–H, a peak at 1596 cm^–1^ indicates C=C, a band between 1400–1300 cm^–1^ shows C–F bonds, and a peak at 1056 cm^–1^ represents C–O.

In the second stage of synthesis (from FOH to FOH-N_3_), the separation of the fluorine groups and the subsequent introduction of the N_3_ groups led to the formation of a characteristic, intense peak at 2123 cm^–1^, corresponding to the “–N–N=N–” bond.

However, during the final synthesis stage, the conversion of N_3_ groups to 1,2,3-triazole structures resulted in the disappearance of the peak at 2123 cm^–1^.^38,39^ Additionally, the emergence of new OH groups contributed to a slight intensification of the OH peak at around 3300 cm^–1^.

### 3.2 Characterization of Rigid Polyurethane Foams

The structure of the azide-functionalized substance employed in the current study and the synthesis routes of the obtained products are depicted in Figure 1. The azide functionality has been generated either by a substitution reaction or by direct use of azide functional structures. Here, the FOH compound was synthesized first according to our previous papers, and the fluorine units in the structure were transformed into azide units, with quantitative conversion, through the nucleophilic substitution reaction using NaN_3_. The azide functionality was formed by substituting the para-fluorine atom in the aromatic rings with the azide group, as described above. The transformation from fluorine to azide was quantitative, as determined by ^19^F NMR spectroscopy, which showed that the para-fluorine signal was not present in the structure (Figure 4). Following the azide-functionalized fluorine compound syntheses, a copper-catalyzed azide–alkyne cycloaddition (CuAAC) “click” reaction was performed between the azide-functionalized FOH-N_3_ structure and propargyl alcohol.

A targeted triol structure (FDOH) was realized for the preparation of RPUFs. Synthesis of polyurethane foam is a complex procedure that includes the reaction of water with isocyanate, forming carbamic acid. The acid then turns into carbon dioxide. The carbon dioxide is dispersed in the polymer precursor and increases until it becomes supersaturated, at which point nucleation begins. The good part of this reaction is its formation rate when carried out under the usage of catalysts and a diversity of commercially available materials, mostly polyols, which can give the opportunity to synthesize special polyurethanes having different types of properties. Based on that, one of the most used approaches to synthesize polyurethanes is the one-shot technique, also used in this study. Here, polymeric methylene diphenyl diisocyanate reacted with 400 OH sucrose-based polyether polyol (trade name) and a polyol mixture, in which different ratios of synthesized FDOH were added to form urethane linkages (−NH–C=O).

#### 3.2.1 FTIR Analysis of RPUFs

**Figure 6:**
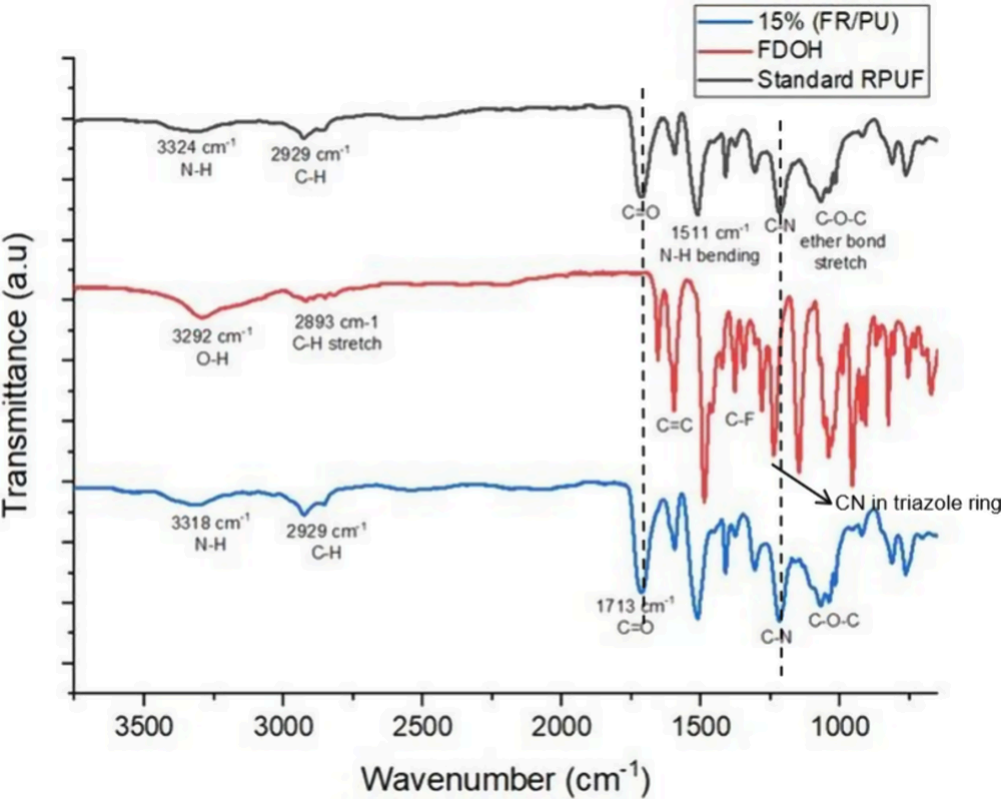
Comparison of FTIR analyses of flame-retardant compounds, neat RPUF, and flame-retardant RPUFs.

In Figure 6, the FTIR spectra of standard and flame-retardant foams are compared with the FTIR spectrum of FDOH. The FTIR spectra of RPUF reveal several characteristic peaks corresponding to different functional groups: the N–H stretching peak was observed around 3300 cm^–1^, C–H stretching at 2929 cm^–1^, C=O stretching at 1713 cm^–1^, N–H bending at 1511 cm^–1^, C–N stretching at approximately 1219 cm^–1^, and C–O (ether) stretching at around 1070 cm^–1^. A new absorption band at 2123 cm^–1^ of azido (−N–N=N−) was observed in FOH-N_3_ and disappeared in FDOH. The C–N band in the triazole ring at 1251 cm^–1^ indicated a successful ‘click’ reaction, as shown in Figure 5. In Figure 6, the −CN band at 1219 cm^–1^ came from −HN-C– stretching in the urethane linkage. With the addition of FRs, there was no significant change except for a slight increase in peak intensities at some peaks. On examination of the spectrum of the FR foam, the absence of any peaks at 2300 cm^–1^ indicates that the −N=C=O groups, which give peaks in this range, did not remain in excess, and it proves that the OH ends of the FDOH successfully reacted with the isocyanates.

#### 3.2.2 TGA Analysis

**Figure 7:**
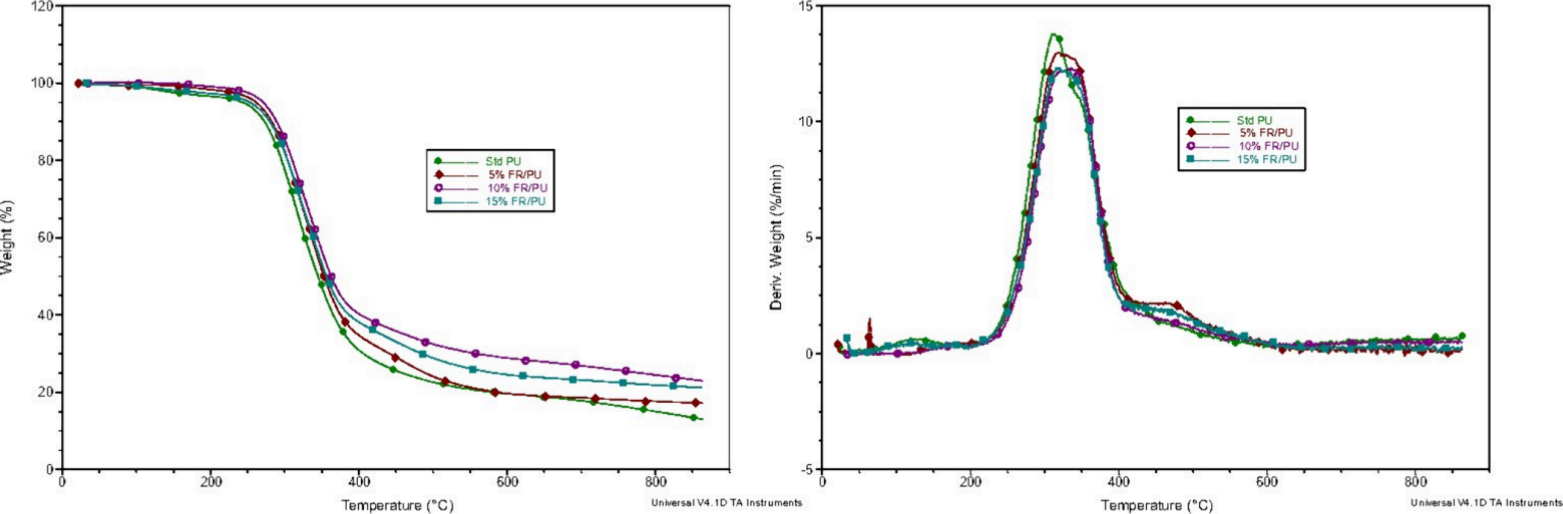
TG (left) and DTG (right) curves of polyurethane foams under a N_2_ atmosphere.

The observed variations in the thermal decomposition behavior of polyurethane foam with varying percentages of flame-retardant additives suggest a nuanced interaction between the additives and the foam matrix. TG and DTG graphics are illustrated in Figure 7. Initially, the addition of flame retardants increases the thermal stability of the foam, leading to increased temperature thresholds when the mass loss is 5 and 50% and the amount of residue when it reaches 800 °C, as shown in Table 3. However, at higher concentrations of flame retardant, a deviation from this trend is observed. In the sample containing 15% flame retardant, the amount of residue and the temperature value at 5% mass loss were below the values of the sample containing 10% flame retardant. Also, in Table 3, according to DTG curves, *T*_max_ and mass loss rate (wt %/min) at *T*_max_ values were given. As we can see from the curves, the mass loss rate decreased with increasing FDOH percentage. From the *T*_max_ value of the foams, it can be seen that a 10% addition is the most ideal.

**Table 3:** Thermal Decomposition Parameters of 0% (Std PU), 5%, 10%, and 15% FR/RPUF (w/w) Samples

sample	*T*_5%_ (°C)	*T*_50%_ (°C)	*T*_max_ (°C)	mass loss rate at *T*_max_ (wt %/min)	residue at 800 °C (wt %)
standard PU	242.81	344.95	312.81	13.75	15.01
5% (FR/PU)	261.71	353.16	318.22	12.97	17.64
10% (FR/PU)	270.89	362.63	335.70	12.31	24.49
15% (FR/PU)	253.29	362.29	319.96	12.34	21.84

**Figure 8:**
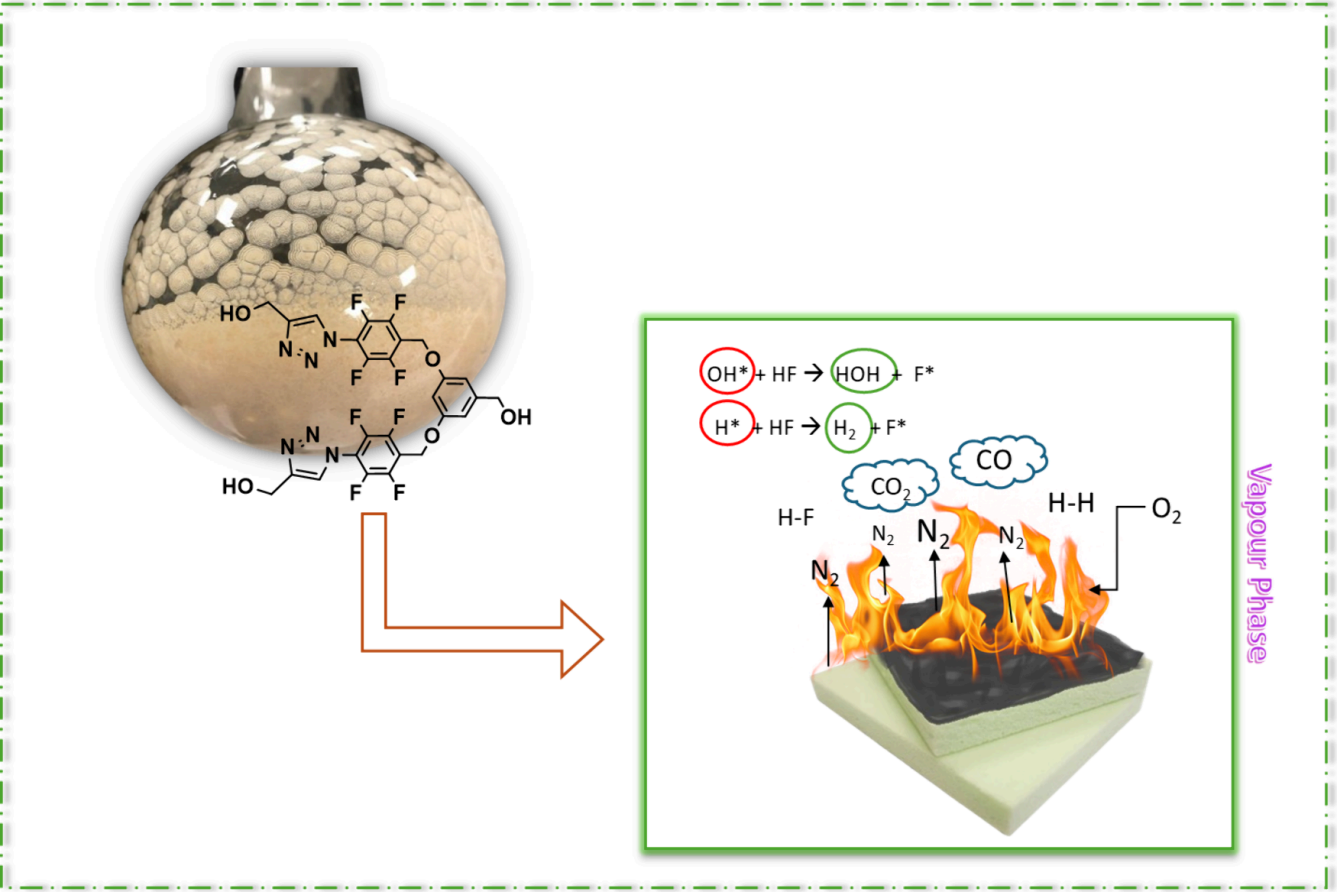
Schematic illustration of the flame-retardant mechanism of FDOH. Rounded flask photograph is courtesy of Merve Nizam. Copyright 2025.

However, while the addition of a flame retardant initially increases the temperature value at 50% mass loss, a 15% addition leads to the stabilization of this temperature threshold. This trend indicates the need to optimize the dose of flame retardant to maintain a balance between the thermal stability and structural properties of the foam.

#### 3.2.3 Limiting Oxygen Index

In this study, the efficacy of incorporating the FDOH material into an RPUF was investigated to improve its flame retardancy. The LOI values of the rigid polyurethane samples are presented in Table 4. The LOI values of all of the flame-retardant RPUF samples are higher than those of the standard (neat) RPUF. Increasing the LOI value demonstrates increased resistance to combustion. The results indicated a notable improvement in the flame retardancy with the addition of FDOH.

**Table 4:** Limiting Oxygen Index (LOI) Values for Polyurethane Foam Samples

sample code	LOI value
standard PU	18.8
5% (FR/PU)	19.3
10% (FR/PU)	19.5
15% (FR/PU)	19.7

#### 3.2.4 Cone Calorimetry Analysis

**Figure 9:**
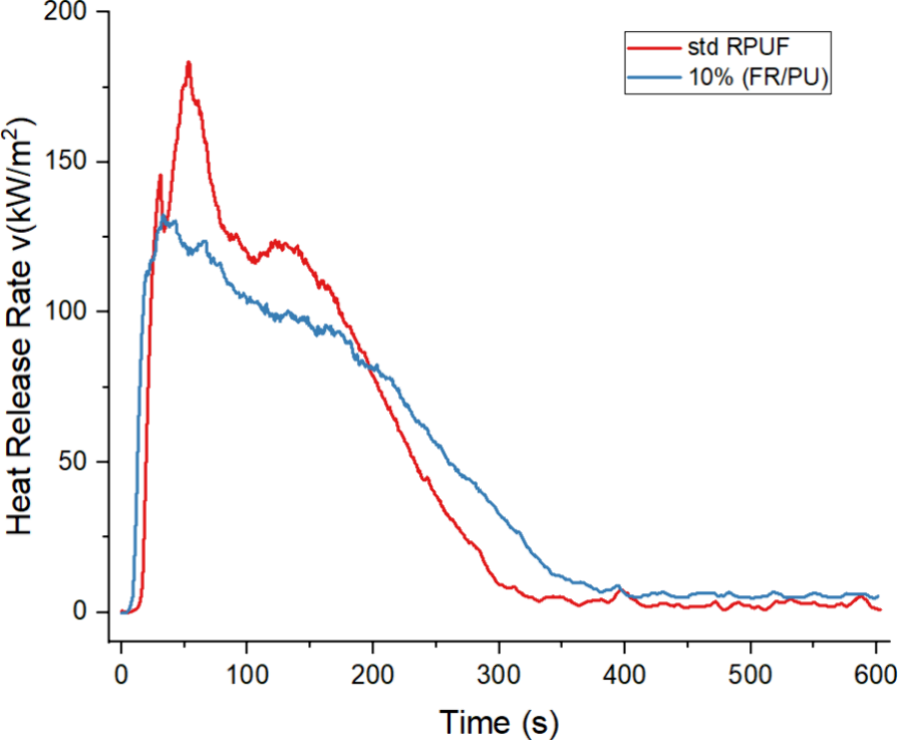
Effect of the flame retardant on the heat release rate.

Cone calorimetry analysis was utilized to gain deeper insights into the impact of FDOH on the flame-retardant behavior of the foams. The cone calorimetry data are recorded in Tables 5 and 6. The rate of heat release is a crucial parameter for assessing combustion trends, correlating with fire spread and flashover occurrences in real fire scenarios.^40^ The heat release rate (HRR) curves of the samples are shown in Figure 9. From Figure 9 and Table 5, it is clearly observed that the standard RPUF burns vigorously and reaches its maximum heat release rate (183.5 kW/m^2^) after 53 s. On the contrary, the 10% foam (FR/PU) exhibits a marked decrease in the maximum rate of heat release, with a decrease of 27.8% compared to the standard RPUF. Standard RPUF gives three district peaks, while flame-retardant RPUF has only one peak point. Moreover, compared to standard foam, flame-retardant foam reaches its maximum value earlier and exhibits a wider heat release property. The table shows the decrease in the heat release rates at the 60th, 180th, and 300th seconds. This demonstrates the effectiveness of the flame retardant.^41^ As can be seen in Table 5, with the addition of 10%FR, a slight increase was observed in the total released heat and average effective combustion heat values. The maximum average rate of heat emission (MARHE) is a metric used to quantify the peak heat release over the duration of a test. It is calculated by dividing the total heat emitted during the test period by the total test time.

**Table 5:** Avr. of Rate of Heat Release (ARHR) of Std. RPUF and 10% (FR/PU)

parameters	ARHR over 60 s (kW/m^2^)	ARHR over 60 s (kW/m^2^)	ARHR over 60 s (kW/m^2^)
standard RPUF	137.7	121.8	88.5
10% (FR/PU)	109.8_(−20.3%)_	103.7_(−14.9%)_	85.6_(−3.3%)_

**Table 6:** Other Cone Calorimeter Data of Std. and 10% (FR/PU)

parameters	PRHR (kW/m^2^)	time of PRHR (s)	THR (MJ/m^2^)	initial/final mass	avr. MLR (g/m^2^s)	EHC (MJ/kg)	MARHE (Kw/m^2^)	CO yield (kg/kg)	CO_2_ yield (kg/kg)
standardRPUF	183.5	53	27.5	17.41/5.57	4.4	23.19	232.22	0.09093	3.57073
10% (FR/PU)	132.5	33	28.1	17.57/6.00	3.7	24.27	205.53	0.06233	3.02533

The addition of a flame retardant was expected to reduce this value, and a decrease from 232.22 to 205.53 kW/m^2^ was observed. This indicates that the flame retardant is effective.^42^ The addition of the flame retardant significantly increased the final mass value while reducing the average mass loss rate from 4.4 to 3.7 g/m^2^s. This can be attributed to the flame retardancy mechanism of FDOH and the formation of a char layer during combustion by the flame retardant, which slows the mass loss shown in Figure 8.

**Figure 10:**
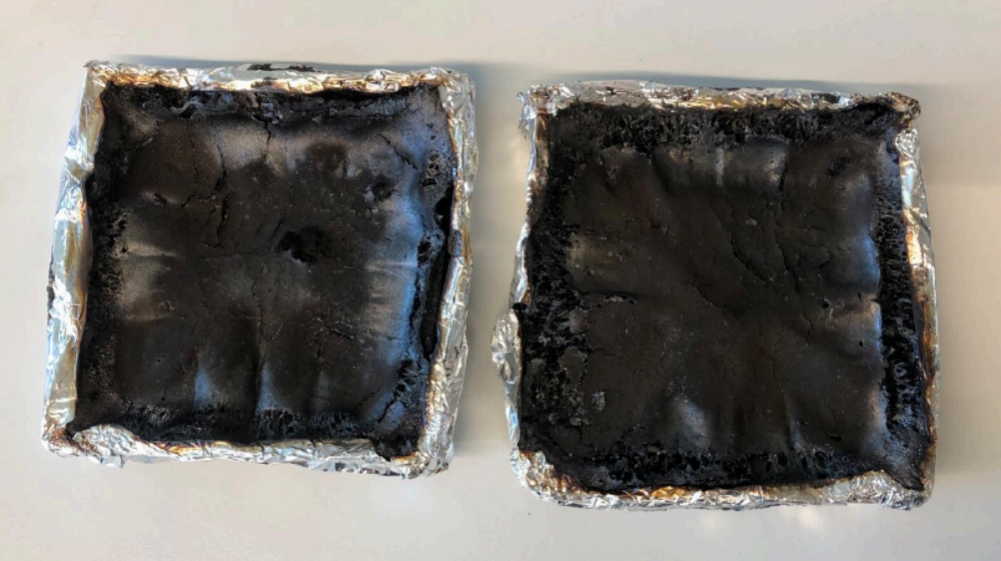
Standard RPU foam (left) and RPU foam containing 10% flame retardant (right) after the cone calorimetry test. Photograph courtesy of Merve Nizam. Copyright 2025.

Toxic gases and smoke, which are as hazardous as fire itself, require the evaluation of parameters such as the CO and CO_2_ yields to assess secondary fire hazards posed by materials. Both the CO and the CO_2_ yields decreased with 10% FR addition. The decrease in carbon monoxide (CO) yield with the addition of the flame retardant suggests that the flame retardant effectively reduces the combustion process and the toxicity of the smoke, thereby reducing the amount of CO produced. The conditions of the RPU foams after the cone calorimeter test can be seen in Figure 10.

#### 3.2.5 Morphological, Mechanical, and Thermoconductivity Properties of RPUFs

**Figure 11:**
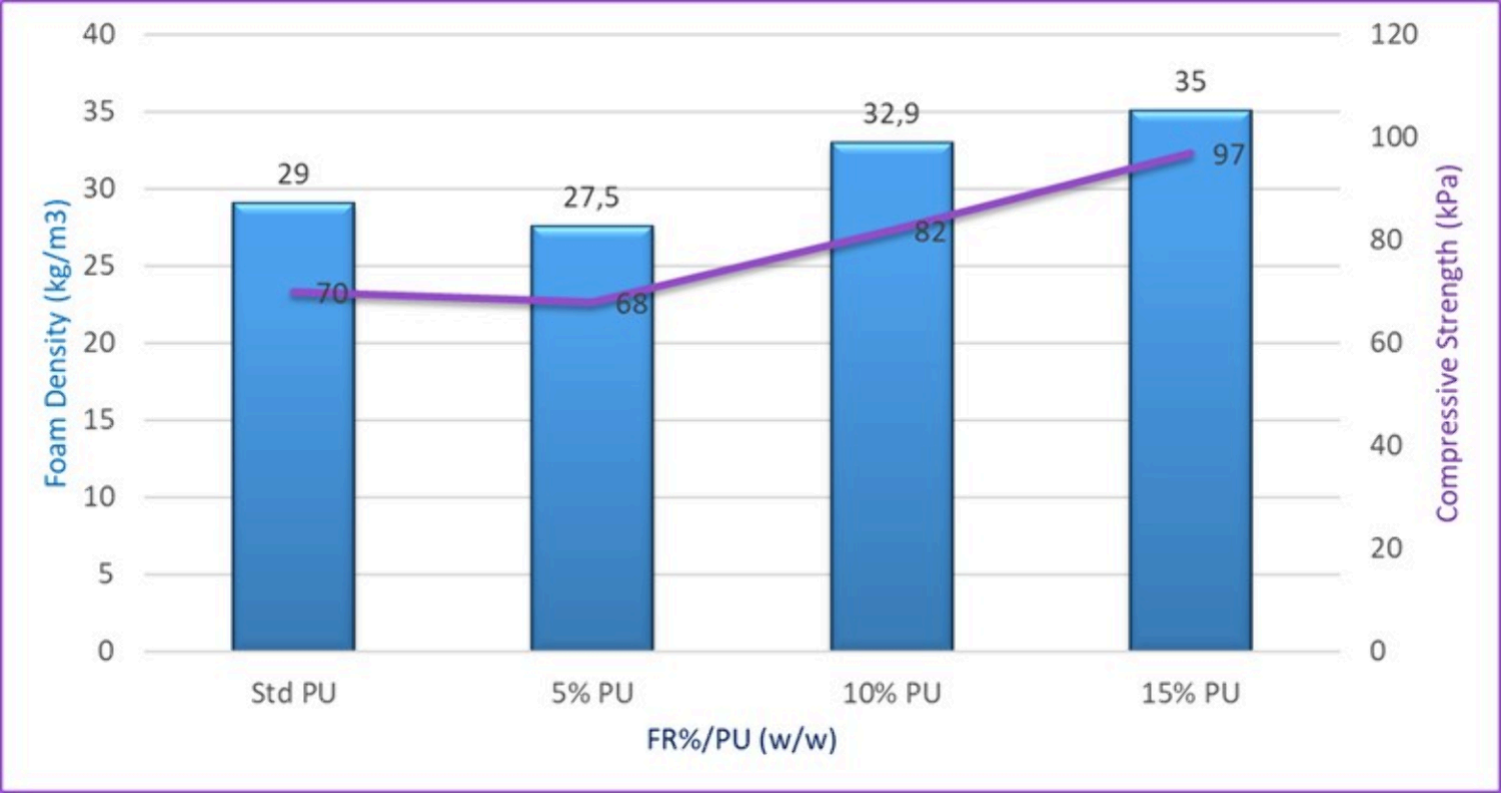
Effect of flame-retardant composition on the compressive strength and foam density.

The compressive strength of RPUF materials is essential for their suitability in the construction industries. The effect of the addition of 5, 10, and 15% FDOH on compressive strength and density was investigated and compared with standard RPUF. As shown in Table 7 and Figure 11, it was observed that increasing FDOH concentrations led to a linear increase in compressive strength and density, except for the 5% (FR/PU) sample. This increase may be due to the aromatic structure of FDOH.^43,44^ The obtained polyurethanes had better properties not only due to the introduction of rigid aromatic units, which caused a higher intramolecular stiffness, but also due to the increase of the intermolecular forces between polymer chains, therefore forming a more uniform and compatible structure.^45^ As the amount of FDOH increases, the −NH and −OH groups in the structure also increase. This causes the formation of hydrogen bonds and cross-linking between the chains. In the case of the sample of 5% (FR/PU), the slight decrease in compressive strength compared to standard PU foam could be attributed to factors such as cell collapse, cell wall rupture, and an increase in average cell size.^46^ The thermal conductivity of the foam without any flame retardant is recorded at 0.02709 W m^–1^ K^–1^. As the flame-retardant content increases to 5, 10, and 15%, a consistent decrease in thermal conductivity is observed, with values of 0.02676, 0.02672, and 0.02658 W m^–1^ K^–1^, respectively. Standard RPUF is a widely used insulation material in construction because of its excellent thermal insulating properties. Naturally, it has a low thermal conductivity value. It is essential that the additives introduced into the foam do not compromise its thermal insulation properties. According to the results obtained, it can be observed that the flame-retardant material does not affect the thermal insulation of the rigid foam; in fact, it may even slightly enhance its insulating capabilities. Additionally, it is noted that it enhances the mechanical strength and density of the foam.

**Table 7:** Comparison of Mechanical and Thermal Properties with and without FR

sample code	density(kg/m^3^)	compressive strength at 10% strain	compressive strength (kPa)	thermal conductivity (W m^–1^ K^–1^)
standard PU	29.0	68	70	0.02709
5% (FR/PU)	27.5	66	68	0.02676
10% (FR/PU)	32.9	79	82	0.02672
15% (FR/PU)	35.0	93	97	0.02658

**Figure 12:**
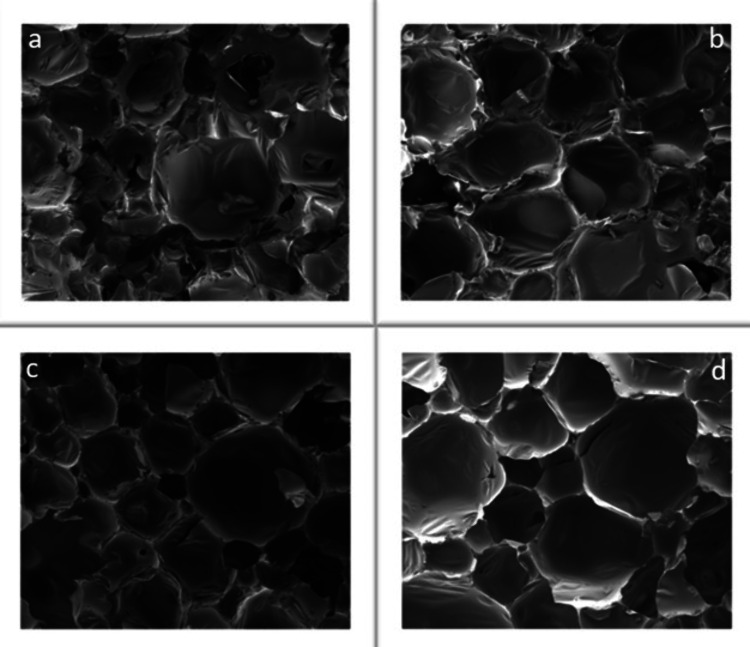
Scanning electron microscopy (SEM) results for FR%/PU samples at 400 μm: (a) Standard PU, (b) 5% FR/PU, (c) 10% FR/PU, and (d) 15% FR/PU.

Cell morphologies of the RPUF samples were investigated with an SEM analyzer, as seen in Figure 12. Normally, RPUFs consist of a high percentage of closed cells.

When SEM analyses of rigid PU foams are examined at 400 nm, we can observe that the closed-cell structure in the standard (blank) foam appears irregular and shapeless. Although it can be observed that the closed-cell structure of all flame-retardant foam samples remains unaffected, as we cross from 5 to 10 and 15%, there is a slight inconsistency and increase in cell diameters. Preservation of the closed-cell structure in RPUFs prevented any detrimental effects on their mechanical and thermal properties. The results of the thermal conductivity and compressive strength measurements further corroborate this.

#### 3.2.6 Contact Angle Measurements

**Figure 13:**
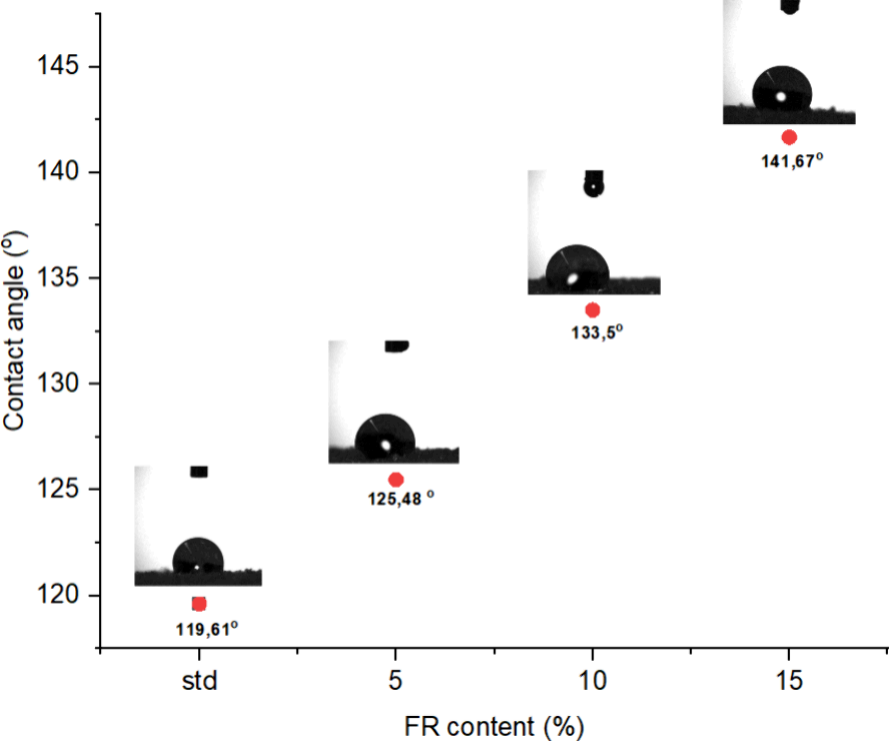
Contact angle changes with an increasing FR content.

The contact angle is the angle formed when a liquid comes into contact with a surface. A higher contact angle means that the liquid adheres less to the surface and gathers more in a spherical shape. This phenomenon indicates that the material is hydrophobic. The higher the contact angle, the more hydrophobic the material is. The results of the contact angle measurement are shown in Figure 13. The addition of 5, 10, and 15% flame retardant to the RPUF caused an increase in contact angles compared to the standard foam sample without flame retardant. Specifically, the contact angles were measured as follows: for standard rigid foam, it was 119.61°, for 5% FRPU, it increased to 125.48°, for 10% FRPU, it further increased to 133.50°, and for 15% FRPU, it reached 141.67 degrees. The main reason for the increase in the outcome values obtained is the presence of fluorines in its structure and an increase in its concentration. C–F bonds are stable and nonpolar bonds.

Their nonpolar nature prevents them from forming weak interactions with polar molecules, such as water. This contributes to the hydrophobic properties of the C–F bonds. Fluorine groups tend to separate and orient toward the surface.^24^

## 4 Conclusions

In this study, the thermal insulation properties and flame retardancy behaviors of the FDOH compound on RPUF were examined. To obtain the FDOH compound, three reaction stages were followed: The FOH compound was synthesized as reported in the literature, the azidization reaction of FOH was realized at para positions, and finally, the FDOH compound was successfully synthesized through a copper-catalyzed azide–alkyne cycloaddition reaction. In RPUF samples, the amount of polyol was reduced while increasing the flame-retardant quantities, whereas the amount of isocyanate remained constant.

When the FDOH mixture containing OH groups reacted with isocyanates, it showed a reactive flame-retardant effect. The absence of the peak corresponding to the −NCO group in the FTIR analyses of the foam samples confirmed this reaction. In general, FTIR spectra have provided valuable information regarding the molecular composition and structural properties of the compound, laying the foundation for further characterization and understanding of its chemical behavior.

While closed-cell structures, which provide thermal insulation properties to the foam, were not compromised by the increase in the percentage of flame retardant, there was a slight increase in the cell size with a 15% addition. However, when looking at the thermal conductivity values, all values remained almost the same as the standard foam’s value of 0.02709 W m^–1^ K^–1^, demonstrating excellent thermal insulation behavior. Moreover, small increases in compression strength and density values were observed with an increasing percentage of flame retardant. Although the LOI values did not increase as much as expected, the cone calorimetry analysis results are promising. In conclusion, this triazole-structured fluorine-containing flame retardant is a novel additive that has not been synthesized or tested on RPUF. In the future, its flame-retardant effect can be enhanced on polyurethane with other additives, used to impart flame retardancy to different polymeric materials, or used and diversified for entirely different purposes.
